# Alpha-1 antitrypsin deficiency-associated liver disease: From understudied disorder to the poster child of genetic medicine

**DOI:** 10.1097/HC9.0000000000000699

**Published:** 2025-04-14

**Authors:** Malin Fromme, Fabienne Klebingat, Paul Ellis, Pavel Strnad

**Affiliations:** 1 Medical Clinic III, Gastroenterology, Metabolic Diseases and Intensive Care, University Hospital RWTH Aachen, Health Care Provider of the European Reference Network on Rare Liver Disorders (ERN RARE LIVER), Aachen, Germany; 2 School of Health Sciences, University of Birmingham, Birmingham, United Kingdom

**Keywords:** fibroscan, liver cirrhosis, liver fibrosis, Pi*S, Pi*Z, *SERPINA1*

## Abstract

Alpha-1 antitrypsin deficiency (AATD) constitutes an inborn disorder arising due to mutations in alpha-1 antitrypsin (AAT), a secreted protease inhibitor produced primarily in hepatocytes. It leads to diminished serum AAT levels, and this loss-of-function predisposes to chronic obstructive pulmonary disease and lung emphysema. The characteristic Pi*Z mutation results in hepatic Z-AAT accumulation. In its homozygous form (Pi*ZZ genotype), it is responsible for the majority of severe AATD cases and can cause both pediatric and adult liver disease, while the heterozygous form (Pi*MZ) is considered a disease modifier that becomes apparent primarily in the presence of other comorbidities or risk factors. In the current review, we collate conditions associated with AATD, introduce typical AAT variants, and discuss our understanding of disease pathogenesis. We present both cross-sectional and longitudinal data informing about the natural disease history and noninvasive tools that can be used for disease stratification as well as a basis for disease monitoring. Given that AATD-associated liver disease is highly heterogeneous, we discuss the risk factors affecting disease progression. While the loss-of-function lung disease is treated by weekly intravenous administration of purified AAT, recombinant modified AAT and oral protease inhibitors are currently in clinical trials. Among the liver candidates, small interfering RNA fazirsiran efficiently suppresses AAT production and is currently in phase 3 clinical trial, while several other genetic approaches, such as RNA editing, are at earlier stages. In summary, AATD represents a systemic disorder increasingly seen in the hepatologic routine and requiring thorough interdisciplinary care, since the currently ongoing clinical trials often address only one of the organs it affects.

## INTRODUCTION

**FIGURE 1:**
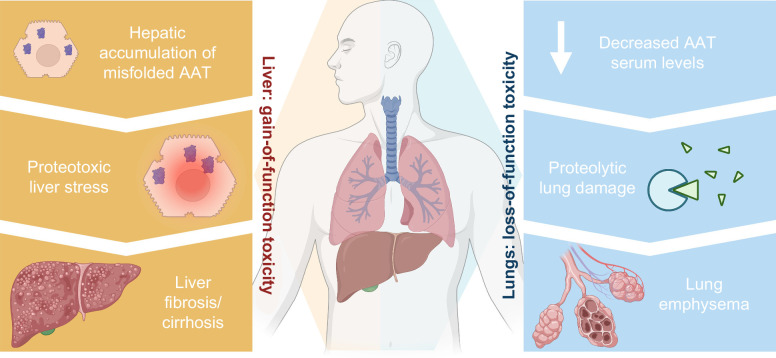
AATD disease pathomechanism with hepatic gain-of-function and pulmonary loss-of-function toxicity. Abbreviations: AAT, alpha-1 antitrypsin; AATD, alpha-1 antitrypsin deficiency.

Alpha-1 antitrypsin (AAT), also known as *ser*ine *p*roteinase *in*hibitor A*1* (*SERPINA1*), counteracts the enzymatic function of multiple proteases such as neutrophil elastase (NE) or proteinase 3, thereby protecting the tissues from excessive degradation.^1^^–^^3^ It constitutes an acute-phase protein produced primarily in the hepatocytes that secrete it into the bloodstream via the endoplasmic reticulum (ER).^1^^,^^4^ Inherited mutations in *SERPINA1* lead to a misfolding and a rapid polymerization of AAT in the ER of hepatocytes and decrease its secretion into the blood, thereby giving rise to the genetic disorder termed alpha-1 antitrypsin deficiency (AATD) (Figure 1).^1^^,^^5^ AATD represents one of the most common, potentially lethal genetic diseases and is characterized by increased proteolytic lung injury (“loss-of-function toxicity”) as well as hepatotoxic liver damage (“gain-of-function toxicity”) that arises due to accumulation of misfolded, often polymerized protein, in the ER of hepatocytes and the corresponding degradation pathways (Figure 1).^4^^,^^6^ More than 200 variants of *SERPINA1* have been described and are further subclassified based on their migration in the electric field. For example, Pi*M refers to “normal,” Pi*F to fast, and Pi*S/Pi*Z to slow/very slow migrating protein, respectively.^7^ The most common mutation leading to severe AATD results from the substitution of a single amino acid, Glu342Lys (Z allele) (Table 1). The homozygous genotype Pi*ZZ is present in 1 of 2000 persons of European descent, while the heterozygous genotype Pi*MZ can be found in 1 of 30 persons of European descent. A milder deficiency caused by the S allele is the result of a different amino acid replacement, Glu264Val, present in 1 of 4 persons in the Iberian Peninsula (Table 1).^7^ Although relatively rare, Pi*ZZ genotype constitutes the predominant cause of severe AATD and the most common cause of AATD-associated lung disease and liver disease.^1^^,^^8^

**TABLE 1:** Overview of characteristic AATD variants with their molecular effects and associated disease phenotypes

Variant	Mutation	Epidemiology	Molecular basis	Lung risk	Liver risk
Z	Glu342Lys	1:27 (UK), 1:80 (US)	Intracellular degradation and polymerization of AAT →low serum levels	Strong predisposition (Pi*ZZ), mild predisposition (Pi*MZ)	Strong predisposition (Pi*ZZ), mild predisposition (Pi*MZ)
S	Glu264Val	1:5 (southern Europe), 1:30 (US)	Misfolding and reduced secretion of AAT →reduced serum levels	Moderate predisposition (Pi*SZ), Minimal risk (Pi*SS)	Moderate predisposition (Pi*SZ), Minimal risk (Pi*SS)
F	Arg223Cys	Rare (<1:1000)	Slow formation of polymers	Risk for lung emphysema in compound heterozygous (Pi*FZ)	Unknown, probably primarily related to the accompanying mutation
I	Arg39Cys	Rare (<1:1000)	AAT misfolding →reduced serum levels	No clear phenotype	No clear phenotype
Q0	Lys217 stop codon (Q0_bellingham_); Δ1bpTyr160 (Q0_granitefalls_); Δ2bpLeu318 (Q0_hongkong_)	Rare (<1:1000 per variant)	Absence of AAT in serum with/without production of AAT protein in hepatocytes	Very strong predisposition to lung emphysema in homozygotes and compound heterozygotes	Variable phenotype, depending on whether a protein is produced and how toxic it is
M_malton_	Phe52del	Most common rare deficiency allele in Sardinia (∼1:365);^5^ rare otherwise (<1:1000)	Intracellular degradation and polymerization of AAT →low serum level	Risk of disease in homozygotes	Risk of disease in homozygotes
Pittsburgh	Met358Arg	Ultra rare (<1:10000)	Function altered to an antithrombin	Fatal bleeding disorder	

Abbreviation: AATD, alpha-1 antitrypsin deficiency.

Given that AATD is defined by an impaired production or secretion of AAT, measurement of AAT serum levels constitutes an easy and cost-effective screening method.^9^ At the same time, it is useful for assessing the severity of AATD in case of rare, poorly characterized mutations since normal or slightly diminished AAT serum levels suggest no or only minimal impairment and vice versa.^10^ A cut-off of 50 mg/dL is used as a triage tool that raises suspicion of severe AATD. Since AAT can double during inflammatory states, such as pulmonary exacerbations, testing in an infection-free interval is recommended.^9^ As the next step, phenotyping can be used when a quick decision is needed, whereas genotyping should be performed for a definitive diagnosis.^11^ The former assesses the migration of AAT in an electric field and cannot distinguish between variants showing similar behavior (such as Pi*M and Pi*MMalton, Pi*Q0 variants, etc.). An administration of AAT augmentation presents another phenotyping challenge. The routine genetic resting typically relies on PCR/multiplex PCR, which is compared with AAT serum levels as a plausibility check. If the results are inconclusive, a sequencing is performed.^12^

## LUNG DISEASE AND OTHER AATD-ASSOCIATED CONDITIONS

AAT inhibits proteases such as NE that otherwise cleave many structural as well as immune proteins. In AATD, decreased and/or functionally impaired AAT fails to fulfill this task (“loss-of-function” mechanism), which becomes particularly detrimental in various stress situations such as smoking or microbial infection that lead to an increased release of proteases including NE.^3^^,^^13^^,^^14^ Moreover, other lung proteases, such as metalloproteases and cysteinyl proteases, are induced and activated by NE.^15^ To make things worse, stress situations may lead to the inactivation of AAT due to oxidation, proteolytic cleavage, and polymerization.^16^ In addition, polymerized Z-AAT acts as a potent neutrophil chemoattractant with proinflammatory effects.^16^ Thus, AATD overall leads to unopposed protease activity, increased number of neutrophils, and development of structural damage in the lungs,^1^ but also increases susceptibility to autoimmune disorders.^17^^,^^18^

Clinically, Pi*ZZ individuals show ~85% decreased AAT serum levels, leading to a strong predisposition to a lung disease that manifests as early-onset, predominantly basal, but also panlobular lung emphysema and/or chronic obstructive pulmonary disease (COPD). In addition to the standard COPD care (ie, inhalation treatment, long-term oxygen in advanced disease, etc.), it can be treated with weekly infusion of plasma-purified human AAT, termed augmentation therapy. Notably, augmentation therapy is not reimbursed in all (European) countries. An observational study from a large European Alpha-1 Research Collaboration (EARCO) patient registry showed that among Pi*ZZ patients, emphysema and COPD were the most frequent lung diseases (57.2% each), followed by bronchiectasis (22%).^19^ Advanced age, male sex, exacerbations, increased platelets and neutrophils, augmentation therapy, and lower AAT serum levels were associated with worse forced expiratory volume in one second (FEV_1_).^19^

Normal to slightly decreased AAT serum levels are found in Pi*MZ subjects. Although population-based studies found no or minimally increased risk for COPD/lung emphysema, Pi*MZ individuals seem to be predisposed to lung disease when additional risk factors such as smoking or susceptible genetic background are present.^1^^,^^20^^–^^22^ 1:600 persons of European descent are affected by the compound-heterozygous genotype Pi*SZ, which is characterized by intermediate AAT serum levels and a moderate risk for AATD-related lung disease.^23^ The Pi*SS and Pi*MS genotypes are clinically less relevant risk factors for the development of lung or liver disease.^24^

Smoking has been shown to be an important risk factor contributing to earlier and more severe lung disease in AATD, as oxidants in cigarette smoke further incapacitate Z-AAT by oxidizing its active-site methionines and increasing polymerization leading to a lack of NE inhibition.^25^^,^^26^ Early diagnosis is essential since it often triggers lifestyle changes, such as increased exercise and smoking cessation. The latter is important since augmentation therapy is reserved only for non-smokers. Given the negative impact of infections, vaccination against respiratory infections is also warranted.^9^^,^^27^

Though the aim of most treatments for AATD-related lung disease is to delay the development of pulmonary emphysema, surgery may be required for those with advanced disease. Surgical options for AATD lung disease include (a) lung volume reduction surgery and (b) lung transplantation.

Lung volume reduction surgery (LVRS) aims to reduce the impact of hyperinflated areas of the lung, either by surgical removal of hyperinflated lung tissue or by insertion of one-way valves that allow trapped air to escape. Evidence is limited for both therapies. In a recent case series of 30 patients with AATD, lung volume reduction using endobronchial valves of the either lower lobes, or middle lobe was shown to be safe with improvements in lung function, 6-minute walk distance and quality of life.^28^ Other studies of surgical LVRS have shown lower rates of success compared to those without AATD.^29^ The consensus is that LVRS, as with non-AATD emphysema, should be reserved for carefully selected patients with heterogenous patterns of lung emphysema.

Lung transplant is indicated in those patients with advanced lung disease. AATD is the fourth leading cause of lung transplantation worldwide, behind cystic fibrosis, interstitial pulmonary fibrosis, and usual COPD, and accounted for 4.9% of lung transplantations between January 1995 and June 2017.^30^ A greater proportion of patients with AATD-related lung disease undergo lung transplantation compared to those with usual COPD, accounting for ~7%–13% of severe AATD cases.^31^^,^^32^ This may reflect their younger age and lower comorbidity burden relative to usual COPD.^33^ Despite being a COPD subset, AATD differs significantly, with transplant candidates typically being younger and having less cigarette exposure.^34^ At referral, AATD patients show similarly poor FEV_1_ and worse diffusion capacity of the lungs for carbon monoxide than usual COPD.^31^

In addition to existing guidelines, a recent Delphi survey between EARCO members provides suggestions for the management of AATD-related lung disease.^35^ An annual spirometry assessment was endorsed for individuals with stable lung disease, whereas a more frequent evaluation was recommended for those with worsening symptoms or spirometric parameters. Additional measures, such as body plethysmography and diffusion capacity, are useful for a comprehensive assessment of lung function and in determining those who may benefit from LVRS. While CT-densitometry is considered useful for emphysema quantification, more research is needed before routine use can be recommended.^35^

**FIGURE 2:**
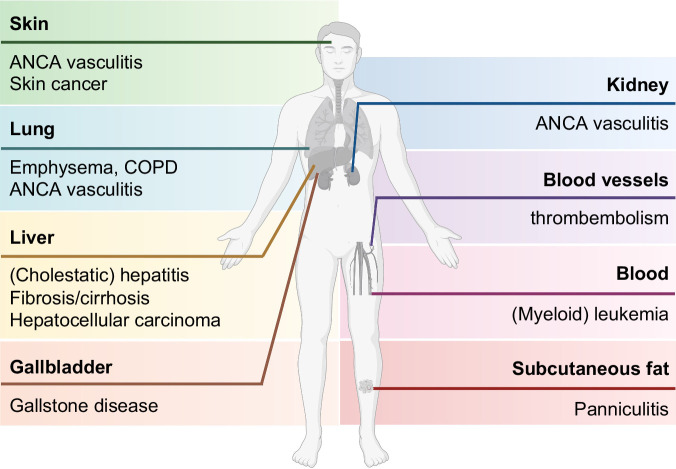
AATD-associated conditions and their clinical presentation. Abbreviations: ANCA, antineutrophil cytoplasmic antibody; COPD, chronic obstructive pulmonary disease.

While lung and liver affections are prevalent in AATD and are the leading causes of AATD-related mortality, multiple other conditions are associated with AATD (Figure 2) and collectively demonstrate the complex biological functions of AAT. Panniculitis refers to inflammation of subcutaneous fat that becomes evident as subcutaneous nodules and the presence of neutrophil infiltrates in the subcutis. Its incidence in AATD has been estimated to be 0.1%–0.9%, but due to the lack of specific diagnostic algorithms in dermatological guidelines, the true prevalence remains unknown.^36^^–^^38^ However, it can lead to large, life-threatening wounds, especially in the presence of concomitant systemic illness.^38^^–^^41^ Most cases are associated with the Pi*ZZ genotype, and dapsone represents the first line of treatment. However, intravenous AAT augmentation therapy may become necessary in resistant or rapidly progressive cases.^38^

Antineutrophil cytoplasmic antibody–associated vasculitis, skin cancer, and (myeloid) leukemia are more frequent in AATD subjects.^42^^–^^45^ The latter might be both due to expression of the mutated AAT in the myeloid lineage as well to manifold effects that extracellular AAT exerts on this cell population.^46^ In Pi*MZ individuals, an increased incidence of gallstones has been reported.^22^ Independent studies also indicated that Pi*ZZ subjects harbor an increased risk of venous thromboembolisms (Figure 2).^47^^,^^48^

## PATHOPHYSIOLOGY OF AATD-ASSOCIATED LIVER DISEASE

AATD serves as a model hepatocellular proteostatic disorder and analyzes of AAT mutations and their fate are shaping our understanding of handling of misfolded secretory proteins.^49^^–^^51^ This includes insights into molecular mechanisms of both key degradation pathways, that is, ER-associated degradation and autophagy.^49^^–^^53^ In all, 70% of the Z-AAT protein (ie, protein resulting from Pi*Z mutation) is degraded, 15% is secreted, and 15% forms insoluble polymers that accumulate in the hepatocytes and lead to proteotoxic stress promoting the development of liver disease.^1^^,^^4^^,^^54^ In clinical routine, the accumulated AAT is visualized by periodic acid-Schiff staining combined with diastase digestion (PAS-D), where it appears as pink globules that can be as large as a nucleus and are located predominantly in the periportal area.^54^ Immunohistochemical staining with a Z-AAT-specific antibody is more sensitive than PAS-D and visualizes even smaller AAT assemblies but is not routinely used.^55^^–^^57^

Monomeric Z-AAT is predominantly removed by ER-associated degradation, while larger Z-AAT polymers are primarily cleared by autophagy.^58^ Notably, experimental studies indicated that subjects with AATD-associated liver disease are less able to degrade the misfolded proteins than individuals without, and patients with advanced liver disease display more AAT aggregates.^55^^,^^56^^,^^59^ In line with that, genetic variants in autophagy genes were proposed as putative modifiers of AATD-associated liver disease phenotype.^60^ The accumulated AAT has several downstream consequences. It causes mitochondrial injury and leads to the solidification of ER, which impairs the mobility of ER proteins.^61^^,^^62^ Ultimately, this observation explains why cells with mutated AAT are hypersensitive against ER stress.^61^

As aggregates may exert their detrimental effects through sequestration of essential proteins,^63^ a recent study used several complementary approaches to isolate AAT inclusions. Endoplasmic reticulum chaperone 78 kDa glucose-regulated protein (GRP78), a key chaperone instrumental for repair and degradation of misfolded ER proteins, was invariably enriched, and treatment with the toxic bile acid cholic acid further increased its abundance within the aggregates.^63^ Therefore, GRP78 sequestration and consecutive depletion might be responsible for the increased stress response that is seen in aggregate-containing cells.^63^^,^^64^

As the currently most advanced effort to uncover the pathomechanism of AATD-associated liver disease, Rosenberger et al subjected formalin-fixed paraffin-embedded sections from human liver biopsies and explants to Deep Visual Proteomics (DVP), a state-of-the-art proteomic approach that combines cell staining with artificial intelligence-based cell shape detection, laser microdissection, and proteomic evaluation. The authors were able to analyze 35 Pi*ZZ individuals and detect more than 5000 proteins per sample. In a subset of them, DVP was combined with proteomic analysis of single cells.^64^ While it unequivocally demonstrated the association of AAT accumulation with ER stress and unfolded protein response, it also provided interesting new insights, such as early peroxisomal upregulation. Moreover, they revealed that AAT accumulation is mostly cell-intrinsic with minimal stress propagation between hepatocytes. It was shown that cells with AAT aggregates proteomically clearly differ from cells without aggregates. Overall, the generated data represent a unique resource that will likely inform the AATD-related research for the years to come.^64^ Finally, Brzozowska et al systematically analyzed somatic mutations in AATD livers and demonstrated that somatic variants in *SERPINA1* are strongly enriched. The detected mutations clustered at the 3′-end of the gene and led to missense or truncation variants. In vitro, these variants suppressed AAT polymerization and ER retention as the likely attempt to escape proteotoxic stress that resulted in clonal expansion of the escaping cells.^65^ These data provide clear evidence that mutations decreasing the AAT mutation-associated proteotoxic stress confer a clear etiology-specific evolutional advantage since such mutations were not found in subjects with hemochromatosis.^65^

## Pi*ZZ-ASSOCIATED LIVER DISEASE

The Pi*ZZ genotype is the leading cause of AATD-associated liver disease. The disease manifests in a biphasic pattern, with the first peak in early childhood and the second peak around the age of 40 years.^66^^,^^67^ The best evidence for pediatric liver disease comes from a Swedish neonatal screening program that identified 120 individuals with Pi*ZZ genotype from 200,000 newborns. Of those, 12% displayed prolonged neonatal jaundice, which constitutes the characteristic disease phenotype. 8% had severe liver disease, and only 2%–3% developed end-stage liver disease requiring liver transplantation.^66^ Despite that, Pi*ZZ represents one of the leading causes of pediatric liver transplantation and offers very good patient and graft survival rates.^68^ Notably, serum liver enzymes were elevated in >50% of the Pi*ZZ newborns and gradually decreased/often normalized during follow-up, resulting in only 12% of individuals with elevated GGT at the age of 18 years.^66^ Of note, pediatric liver disease mostly appears to be cholestatic with prolonged jaundice, ductular proliferation, and even ductopenia in some patients.^54^^,^^69^ While predictive biomarkers are lacking and the prognostication, therefore, relies on rather generic signs of advanced liver disease with signs of portal hypertension, a recent study indicated that GGT levels together with high circulating Z-AAT polymers might indicate a future progressive disease course.^70^

A major caveat when studying Pi*ZZ-associated phenotype is the fact that the majority of Pi*ZZ subjects remain undiagnosed. Because of that, population-based databases possessing AAT genotyping in all participants, such as the United Kingdom Biobank (UKB), represent a unique resource. UKB recruited ~500,000 individuals and collected a large amount of healthcare-related data due to linkage to medical health records that include ICD10 codes.^24^ UKB contains 138 Pi*ZZ, 864 Pi*SZ, and 17,006 Pi*MZ individuals, as well as 422,506 subjects without major AAT mutations termed as noncarriers. Notably, mean ALT/AST serum levels were higher in all AATD genotypes compared to noncarriers. However, it is important to note that the vast majority of Pi*ZZ carriers displayed normal AST and ALT levels, which impairs their detection in a routine hepatologic screening.^24^

Despite the relatively discrete differences in serum transaminases, the ICD10 codes for liver fibrosis/cirrhosis (K74.0-2 and K74.6) were 20 times more common in Pi*ZZ individuals compared to noncarriers (adjusted odds ratio [aOR], 21.7).^24^ The OR for primary liver cancer was even higher (aOR, 44.5), while a study from the Swedish National AATD register detected a >20 times increased adjusted HR.^24^^,^^71^

These data are in line with a second cohort stemming from the European AATD liver study group that recruited nearly 600 Pi*ZZ participants who were all subjected to several noninvasive liver fibrosis tests (NITs).^24^^,^^72^ Based on the applied NIT, significant liver fibrosis corresponding to *F*≥2 on a 0–4 scale was detected in 20%–36% Pi*ZZ individuals, and signs of advanced fibrosis were 9-fold to 20-fold more common in Pi*ZZ subjects than noncarriers. Controlled attenuation parameter (CAP) as a surrogate marker for liver steatosis suggested severe steatosis in 39% of Pi*ZZ carriers versus 31% of controls, and CAP values were significantly higher in Pi*ZZ subjects than in noncarriers.^72^

While histological data are somewhat limited, they confirm and validate the findings described above. Clark et al^55^ examined 94 biopsied non-cirrhotic Pi*ZZ adults and detected significant liver fibrosis (ie, *F*≥2 on METAVIR 0–4 scale) in 35% of all Pi*ZZ patients. Based on PAS-D staining, AAT inclusions were seen in 95% of all individuals. While GGT was the best noninvasive parameter to detect significant liver fibrosis, liver stiffness measurement (LSM) by vibration-controlled transient elastography (VCTE) was superior to recognize advanced liver fibrosis (ie, *F*≥3).^55^ High amount of liver fibrosis was also seen in a second US Pi*ZZ biopsy cohort, while a European cohort confirmed the ability of LSM to reflect histological liver fibrosis.^57^^,^^73^

While the above-described studies were cross-sectional, a European AATD consortium recently presented a longitudinal cohort assessing the predictive usefulness of NITs collected during a baseline examination.^74^ The study comprised 737 Pi*ZZ adults from 25 different centers who were followed up for a median time of 3.5 years and demonstrated that lung and liver disease constituted the leading causes of death.^74^ It revealed an excellent predictive utility of NITs to predict major adverse liver outcomes that were defined as first hepatic decompensation, liver transplantation, or liver-related death.^74^ The most accurate 5-year prediction was achieved by LSM via VCTE (AUC, 0.95), followed by aspartate aminotransferase-to-platelet ratio index (APRI; AUC, 0.92) and Fibrosis-4 index (FIB-4; AUC, 0.90). In contrast, baseline lung parameters displayed only a moderate predictive utility for lung-related endpoints within 5 years (FEV_1_ AUC, 0.76). Notably, many of the assessed individuals suffered liver-related death despite an age that would qualify them for liver transplantation.^74^ While liver transplantation offers good survival rates in carefully selected Pi*ZZ adults, these findings suggest that many of them do not reach it, possibly due to (lung) comorbidity or a rapid pace of liver decompensation.^74^^,^^75^ In line with this hypothesis, an observational cohort study of patients in the Mayo Clinic Healthcare System suggested that AATD-associated liver disease progresses more rapidly in patients without comorbid lung disease.^76^ Not unexpectedly, individuals with AATD-associated liver disease who had no lung disease, were still more likely to undergo liver transplantation compared to those with additional lung disease.^76^ Notably, combined lung–liver transplantation is available only in a few centers worldwide, and subjects with both affected organs need to be sent to such a center.

**FIGURE 3:**
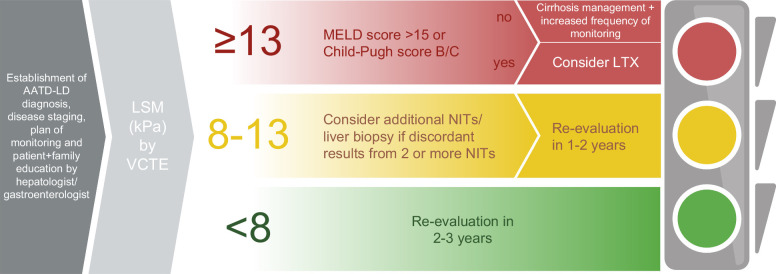
Clinical algorithm for the management of AATD-associated liver disease suggested by Delphi panel study (according to Clark et al).^77^ Abbreviations: AATD-LD, alpha-1 antitrypsin deficiency-associated liver disease; LSM, liver stiffness measurement; LTX, liver transplantation; NIT, noninvasive liver fibrosis test; VCTE, vibration-controlled transient elastography.

The existing study results were mirrored in both an EASL (European Association for the Study of the Liver) guideline as well as a Delphi panel exercise that both suggested VCTE as the preferred NIT for the (initial) assessment of AATD-associated liver disease, although magnetic resonance elastography and enhanced liver fibrosis tests were also considered valuable.^11^^,^^77^ Most panelists agreed that VCTE <8 kPa indicates no/mild fibrosis, VCTE ≥8 kPa clinically significant fibrosis (fibrosis stage ≥F2 on the METAVIR scale) and ≥13 kPa liver cirrhosis.^77^ Based on the EASL guideline, liver biopsy should be considered when a careful noninvasive evaluation remains inconclusive.^11^ Although detailed clinical guidelines for the management of AATD-associated liver disease are still missing, the Delphi panel study suggested a VCTE-based assessment, that is, re-evaluation every 2–3 years in subjects with VCTE <8 kPa versus a more thorough monitoring with management of liver cirrhosis in those with VCTE ≥13 kPa (Figure 3).^77^ Despite the lack of evidence, lifestyle counseling is recommended, given the fact that smoking and obesity have negative effects on the health of AATD patients.^11﻿^

While the liver phenotype of Pi*ZZ individuals is highly variable, the biological factors responsible for this heterogeneity are far from being understood. While genetic variants affecting protein degradation may play a role, the currently available findings need to be replicated in independent cohorts.^59^^,^^60^ Notably, the common *PNPLA3* variant does not seem to play a major role.^78^ Somewhat surprisingly, several independent studies failed to demonstrate an association between alcohol consumption and Pi*ZZ-associated liver phenotype, which might be in part due to the fact that the number of subjects with harmful alcohol consumption was low in these volunteer-based studies.^57^^,^^79^^,^^80^ On the other hand, several independent lines of evidence suggest a detrimental effect of metabolic risk factors, particularly obesity and diabetes, but the magnitude of this effect still needs to be properly evaluated.^55^^,^^57^^,^^72^^,^^74^^,^^81^ Similarly, an increased risk of liver fibrosis in males is well established and is likely related to an increased production of AAT that is driven by testosterone.^54^ Among AATD-specific factors, increased amounts of Z-polymers were found in those with a more advanced liver fibrosis and may predispose to an adverse disease course.^57^^,^^70^^,^^82^

## Pi*MZ-ASSOCIATED LIVER DISEASE

Unlike the disease-causing Pi*ZZ genotype, the heterozygous Pi*MZ genotype displays normal or slightly decreased serum AAT levels and is considered a disease-modifying factor.^54^^,^^56^ While Pi*MZ individuals in the UKB presented with a 1.7 times increased risk of liver fibrosis/cirrhosis, the simultaneous presence of “second hits,” that is, risk factors or other liver disorders (such as steatotic liver disease, alcoholic liver disease, cystic fibrosis) substantially increases the risks.^24^ In line with that, a large genome-wide association study showed that the Pi*Z variant is associated with 2–3 times increased odds for alcohol-associated liver disease–related/metabolic dysfunction–associated steatotic liver disease–related cirrhosis, thereby surpassing the risks conferred by established genetic liver disease modifiers, for example, *PNPLA3* p. Ile148Met (rs738409), hydroxysteroid 17-beta dehydrogenase 13 (*HSD17B13*):T (rs72613567), and *TM6SF2* p. Glu167Lys (rs5854926).^83^ Risk factors for the development of liver fibrosis were found to be the same as in other AATD genotypes: male sex, presence of obesity, and age ≥50 years.^56^ In children, PI*MZ status constitutes one of the best-established genetic factors increasing the risk of cystic fibrosis-associated advanced liver disease.^66^ On the other hand, Pi*MZ individuals do not seem to carry an elevated risk of liver cancer.^54^^,^^84^

Recent studies clearly demonstrate that harboring the Pi*Z allele was significantly associated with an increased probability of liver transplantation and liver-related death.^85^^,^^86^ Notably, among Whites, Pi*MZ subjects constitute up to 10% of liver transplant candidates.^54^ In Pi*MZ patients with liver cirrhosis, hepatic decompensation, and liver-related death were significantly accelerated compared to noncarriers with liver cirrhosis. Thus, Pi*MZ individuals with liver cirrhosis should be seen in a specialized center, and listing for liver transplantation should be performed without delay after the first hepatic decompensation. As a potential underlying mechanism, the hyperinflammation that is often found in decompensated liver cirrhosis increases the production of the acute-phase reactant AAT and thereby increases the proteotoxic ER stress.^54^

Because of the above-described risks, the EASL guideline recommends that Pi*MZ organs should only be considered for living donor liver transplantation when other suitable organs are lacking, and no signs of liver injury are present. Unsurprisingly, livers from Pi*ZZ individuals should not be used as donor organs.^11^

## Pi*SZ-ASSOCIATED LIVER DISEASE

While the Pi*SZ genotype is relatively common (ca. 1:500 in individuals of European descent), it is associated with only ~3 times elevated risk of liver fibrosis but nearly 7 times increased risk of primary liver cancer.^24^ Lung-related risks seem to be analogous, and the Pi*SZ genotype is therefore sometimes described as “intermediate” AATD.^23^^,^^24^

## THERAPEUTIC APPROACHES FOR THE AATD-RELATED LUNG DISEASE AND LIVER DISEASE

In 1987 the Food and Drug Association approved intravenous AAT augmentation therapy with plasma-purified AAT for the treatment of lung disease associated with severe AATD. The approval was based on its ability to restore nearly normal AAT levels rather than clinical endpoints.^87^ Later on, the RAPID trial, a randomized, placebo-controlled trial, proved a significant reduction in the annual rate of lung density loss. A significant effect on FEV_1_, quality of life, or exacerbations of COPD, could, however, not be shown.^88^ Recently, Fraughen et al conducted an observational study evaluating 615 Pi*ZZ individuals from 3 countries and were able to demonstrate a survival benefit in countries administering intravenous augmentations therapy (*p*<0.001). Notably, the improved survival was largely decoupled from FEV_1_ decline except in patients with a Global Initiative for Chronic Obstructive Lung Disease (GOLD) stage 2 lung index, a group commonly outside current augmentation therapy commencement recommendations.^89^ Another study found augmentation therapy was associated with reduced rate of quality of life decline though there was not a signal for mortality benefit.^90^ These data highlight the need for biomarkers of early AATD-associated lung disease to facilitate a timely start of augmentation.

While augmentation treatment primarily targets the loss-of-function lung phenotype, AAT is known to display anti-inflammatory and immunomodulatory properties that may also benefit the liver.^91^ In fact, augmentation treatment was associated with somewhat improved liver injury/fibrosis surrogates and less histological inflammation.^79^ While the data are intriguing, subpopulations with/without augmentation treatment differ by the extent of lung injury, and prospective studies are needed as definitive proof of efficacy﻿.

Several other drug candidates for the AATD-related lung disease are currently in clinical trials (Table 2). Among them, INBRX-101 constitutes a recombinant fusion protein containing 2 AAT molecules linked to a Fc region of human IgG that just completed a phase 1 study. Weekly INBRX-101 infusions were able to maintain sufficient serum AAT levels with a good safety profile. Modeling of AAT serum levels suggested that infusions every 3–4 weeks might be sufficient with the corresponding improvement in quality of life.^95^

**TABLE 2:** Overview of available/currently tested therapeutic options for AATD-related lung disease and liver disease

Target organ	Intervention	Approved/clinical study (phase)	Effect and selected data
Lung	Augmentation therapy with plasma-purified AAT (i.v. infusion)	Approved	Decreases loss of lung density, no effect on FEV_1_ (eg, RAPID trial^84^)
Lung	Recombinant human AAT-Fc fusion protein with a longer half-life (i.v. infusion)	Phase 1	Prolonged serum half-life less frequent dosing and a lower burden on patients expected (eg, INBRX-101^88^)
Lung	Oral NE inhibitor	Phase 2	Significant reduction in NE activity and the NE-specific fibrinogen cleavage product A-alpha-Val360, improved patient-reported outcomes (eg, Alvelestat^89^)
Lung	Inhaled AAT	Phase 3	(eg, “Kamada-AAT”, KB408)
Liver	siRNA hepatocyte-specific delivery, degradation of AAT mRNA	Phase 3	Decline in serum Z-AAT concentration and hepatic Z-AAT accumulation (eg, Fazirsiran^92^^,^^93^)
Liver/both	Folding corrector	Phase 1	(eg, BMN 349)
Both (lung+liver)	RNA editing correction of point mutation via endogenous deaminase	Phase 2	Single dose led to a correction of more than 60% of total AAT, the first reported successful in vivo human RNA editing (eg, WVE-006,^94^ KRRO-110)
Both (lung+liver)	DNA editing	Phase 2	(eg, BEAM-302)

Abbreviations: AAT, alpha-1 antitrypsin; AATD, alpha-1 antitrypsin deficiency; NE, neutrophil elastase; siRNA, small interfering RNA.

Another lung-directed compound is alvelestat (MPH966), an oral NE inhibitor. A phase 2 study showed that alvelestat was safe, well-tolerated, and led to a significant reduction in NE activity and the NE-specific fibrinogen cleavage product A-alpha-Val360 at 120 mg twice daily.^96^ Notably, alvelestat administration was also associated with improved scores in St. George’s Respiratory Questionnaire, which is designed to measure health impairment in COPD patients.

In contrast to AATD-related lung disease, there is no disease-specific therapy available for the liver apart from liver transplantation in end-stage liver disease. Notably, since the majority of AAT is produced in hepatocytes, serum AAT concentrations normalize rapidly after the surgery.^75^^,^^97^ Although a decline in lung function was reported in a few transplanted individuals, the overall risk of lung-related death is low in carefully selected individuals.

Since AATD constitutes a relatively common rare disorder with Pi*Z as the dominant genetic variant, it became the “poster child” for the rapidly developing gene therapies (Table 2). The current surge in clinical trial activity is based on solid experimental evidence, and the candidates can be subdivided based on the targeted molecule (ie, DNA/RNA) as well as a mode of action (ie, virus-based gene addition, CRISPR-based deletion, DNA/RNA editing, RNA interference, etc.).^92^^–^^94^ Among them, the most clinically advanced compound is fazirsiran, an N-acetylgalactosamine-conjugated RNA interference candidate that is specifically delivered to hepatocytes via the asialoglycoprotein receptor.^98^ The design and mode of action are comparable to other U.S. Food and Drug Administration–approved RNA interference compounds, that is, a highly chemically modified dsRNA that is ultimately loaded on the endogenous RNA-induced Silencing Complex that mediates a long-term suppression of the target sequence.^94^^,^^98^ Data from a phase 2, open-label trial (NCT03946449) showed that all treated patients reduced their accumulation of Z-AAT in the liver (median reduction 83% at week 24 or 48) and a similar reduction in serum AAT levels that constituted the primary endpoint.^99^ Treatment was associated with decreased serum liver enzymes.^99^ Recently published data from a placebo-controlled phase 2 trial (NCT03945292) showed a similarly impressive decline in serum Z-AAT concentration as well as hepatic Z-AAT accumulation and further highlighted the safety of fazirsiran. In particular, no adverse events leading to drug discontinuation were reported in both trials and the overall side effect profile was comparable to placebo group.^99^^,^^100^ Pulmonary function tests remained stable in both trials.^99^^,^^100^ Nevertheless, further data are needed to evaluate the long-term lung safety of fazirsiran. Notably, fazirsiran is currently being tested in a worldwide, phase 3, placebo-controlled trial that includes Pi*ZZ subjects ranging from clinically significant liver fibrosis to compensated cirrhosis (NCT05677971).

RNA editing represents a novel gene therapy technique, and AATD spearheads its clinical development. Similar to RNA interference, RNA editing in AATD relies on targeted delivery of nucleic acids into hepatocytes and their subsequent recruitment to endogenous adenosine deaminase acting on RNA enzymes.^92^ Adenosine deaminase acting on RNA then catalyzes the deamination of the target sequence that edits adenosine to inosine. Since inosine is interpreted as guanosine, RNA editing of Pi*Z aims to correct the causative E342K mutation achieving a functional cure and would potentially benefit both the liver and the lungs. In the RestorAARion-2 trial (NCT06405633), 2 Pi*ZZ patients were treated with WVE-006, an N-acetylgalactosamine-conjugated RNA compound.^101^ The efficacy of the approach was monitored primarily by measuring the levels of wild-type AAT protein, termed M-AAT. Based on a press release, a single dose of WVE-006 led to circulating plasma M-AAT of ~7 µM at day 15, that was interpreted as a correction of more than 60% of total AAT that would be consistent with situation seen in patients with the Pi*MZ genotype.^101^ Notably, these data represent the first reported successful in vivo human RNA editing.

While AATD is now the target of an unprecedented number of clinical trials and is instrumental in the development of novel drug classes, further clinical data are urgently needed to define the natural disease course, detect subjects at risk, and facilitate an individualized risk assessment that considers both lung and liver disease.

## CONCLUSIONS

Recent years have seen the transformation of AATD-associated liver disease from a neglected, understudied orphan disease to a poster child of genetic medicine. Despite that, the vast majority of individuals with AATD, even those with Pi*ZZ genotype, remain undiagnosed or are mislabeled as alcohol-associated liver disease or idiopathic cirrhosis.^102^ But as there are now the first therapeutical drug candidates in clinical trials, AATD should increasingly become part of routine hepatologic practice, and a widespread appreciation of this complex disorder with multiple genotypes and two affected organs is essential. The drug development needs to be accompanied by an assessment of the natural disease course and further clinical/mechanistic studies to be able to translate the current efforts into patient management (Table 3).

**TABLE 3:** Key research questions for individuals with AATD and research agenda for the coming years

Pi*MZ	- What “second hits” are leading to liver disease development? - Do (some) patients benefit from “Z variant-targeted” drugs?
Pi*ZZ	- What is the natural disease course, and what factors determine disease progression? - What are the biological mechanisms underlying pediatric and adult AATD-related liver disease? - What endpoints should be used in clinical trials? - What are the “toxic species” in AATD? (ie, the molecular consequences of different AAT assemblies) - What is the best model to study AATD-associated liver disease mechanisms? - Do we need AATD-specific questionnaires to evaluate disease burden?

Abbreviations: AAT, alpha-1 antitrypsin; AATD, alpha-1 antitrypsin deficiency.
